# The Neuroplastin Adhesion Molecules Are Accessory Proteins That Chaperone the Monocarboxylate Transporter MCT2 to the Neuronal Cell Surface

**DOI:** 10.1371/journal.pone.0078654

**Published:** 2013-11-18

**Authors:** Marieangela C. Wilson, Michaela Kraus, Hassan Marzban, Justyna R. Sarna, Yisong Wang, Richard Hawkes, Andrew P. Halestrap, Philip W. Beesley

**Affiliations:** 1 School of Biological Sciences, Royal Holloway University of London, Egham, Surrey, United Kingdom; 2 School of Biochemistry, University of Bristol, Bristol, United Kingdom; 3 Department of Cell Biology and Anatomy and Hotchkiss Brain Institute, University of Calgary, Calgary, Alberta, Canada; NHLBI, NIH, United States of America

## Abstract

**Background:**

The neuroplastins np65 and np55 are two synapse-enriched immunoglobulin (Ig) superfamily adhesion molecules that contain 3 and 2 Ig domains respectively. Np65 is implicated in long term, activity dependent synaptic plasticity, including LTP. Np65 regulates the surface expression of GluR1 receptor subunits and the localisation of GABA_A_ receptor subtypes in hippocampal neurones. The brain is dependent not only on glucose but on monocarboxylates as sources of energy. The. monocarboxylate transporters (MCTs) 1–4 are responsible for the rapid proton-linked translocation of monocarboxylates including pyruvate and lactate across the plasma membrane and require association with either embigin or basigin, proteins closely related to neuroplastin, for plasma membrane expression and activity. MCT2 plays a key role in providing lactate as an energy source to neurons.

**Methodology/Findings:**

Here we use co-transfection of neuroplastins and monocarboxylate transporters into COS-7 cells to demonstrate that neuroplastins can act as ancillary proteins for MCT2. We also show that *Xenopus laevis* oocytes contain endogenous neuroplastin and its knockdown with antisense RNA reduces the surface expression of MCT2 and associated lactate transport. Immunocytochemical studies show that MCT2 and the neuroplastins are co-localised in rat cerebellum. Strikingly neuroplastin and MCT2 are enriched in the same parasagittal zebrin II-negative stripes.

**Conclusions:**

These data strongly suggest that neuroplastins act as key ancillary proteins for MCT2 cell surface localisation and activity in some neuronal populations, thus playing an important role in facilitating the uptake of lactate for use as a respiratory fuel.

## Introduction

The neuroplastins np55 and np65 are immunoglobulin superfamily (IgSF) adhesion molecules enriched at synapses [1]–[4]. They are type 1 membrane proteins and comprise 2 (np55) and 3 (np65) Ig domains respectively, a single membrane-spanning sequence, a short hydrophilic C-terminal intracellular domain and 6 putative sites for N-linked glycosylation [3], [5]. Np65, but not np55, exhibits trans homophilic binding [4], [6]. The neuroplastins show closest homology to CD147/basigin (also termed 5A11, EMMPRIN, HT7, neurothelin, gp42). Together with embigin (gp70), these glycoproteins form a distinct grouping within the IgSF [7]. All three group members, unusually, contain a charged glutamate residue in the transmembrane domain.

Np65 is brain specific and localised to subsets of predominantly forebrain neurones whereas Np55 is present in all brain regions and is also detected in many other tissues [1], [3], [5]. Both neuroplastins are enriched at synapses [1], [2], [4]. Np65, but not np55, is present in the post synaptic density (PSD) and is translocated into the PSD in response to sustained neuronal activity [4].

The neuroplastins mediate several cellular processes including cell-cell adhesion, neurite outgrowth and activity-dependent long term plasticity [4], [6], [8], [9]. For example, neuroplastin antibodies and a neuroplastin-Fc chimeric protein block the maintenance of long term potentiation (LTP) in hippocampal slices [4]. This inhibition of LTP is mediated by activation of p38 MAP kinase resulting in a down-regulation of AMPA receptor GluR1 subunits at the cell surface [10]. Recently np65 has also been shown to bind to and regulate the localisation of α1-and α2-containing GABA_A_ receptor subtypes [11]. The role of np65 in synaptic plasticity and in neurite outgrowth is dependent upon trans homophilic binding between np65 molecules located on pre- and post synaptic membranes or opposing neuronal membranes [4], [6]. However, both neuroplastins promote neurite outgrowth, np55 by binding to and activating the FGF1 receptor and np65 both by trans homophilic binding and by activation of the FGF1 receptor [6], [8], [9].

The brain is crucially dependent not only on glucose, but on monocarboxylates such as lactate and pyruvate as sources of energy [12]. The transport of monocarboxylates across the plasma membrane is dependent upon specific transport proteins, the MCTs. The MCT family comprises 14 members of which MCTs 1–4 mediate the proton-linked transport of monocarboxylates such as lactate, pyruvate and ketone bodies across the plasma membrane [12]. In this report we identify a novel role for neuroplastins in regulating neuronal energy metabolism by acting as an essential accessory protein required for lactate transport into neurons by MCT2. Neurons have been shown to use lactate released by glial cells as an important respiratory fuel and MCT2 has been implicated as the major MCT isoform responsible for this uptake [13]. It is well established that the close homologs of neuroplastins, basigin and embigin, are required for the correct plasma membrane expression and transport function of specific MCTs [13]–[17]. Basigin has been identified as the accessory protein for MCTs 1 and 4, but not MCT2 while embigin is the preferred partner for MCT2 in some tissues [16], [18].

Previously no data have been available on a role for neuroplastins as accessory proteins for MCT2 in the nervous system. Here we demonstrate that neuroplastins are accessory proteins for MCT2, both chaperoning it to the neuronal cell surface and supporting lactate transport by MCT2. We also demonstrate that MCT2 and neuroplastin co-localise in the cerebellum, suggesting a key role for the neuroplastins in supporting MCT2 function in specific neuronal populations.

## Materials and Methods

### Live confocal imaging of cells and fluorescence resonance energy transfer (FRET)

COS-7 cells were maintained in DMEM containing 10% fetal calf serum (FCS), 1× antibiotic/antimitotic (Gibco) and 2 mM L-glutamine. For confocal imaging and FRET, COS-7 cells were transiently transfected with MCT2-ECFP (N-terminal) and np55 or np65-EYFP (C-terminal) tag using Fugene 6 (Roche) 48 h prior to imaging or measurement of FRET. Imaging was performed at 37°C in imaging buffer (136 mM NaCl, 4.7 mM KCl, 1.25 mM MgSO_4_, 1.25 mM CaCl_2_, 2 mM NaHCO_3_, 5 mM Na phosphate, 25 mM Hepes pH 7.4) using a Leica confocal imaging spectrophotometer system (TCS-SP2) attached to a Leica DMIRBE inverted epifluorescence microscope equipped with a 430 nm laser, an argon laser (458, 476, 488, 514 nm lines) and an acousto-optic tunable filter to attenuate individual visible laser lines [16], [19]. Cells were imaged with a 63×1.32 Na oil immersion objective. Quantification of FRET was performed by calculation of the ratio of intensity of the fluorescence emission signal at 480 nm to that at 530 nm when excited at the ECFP excitation wavelength (430 nm) as described previously [16], [19].

### Detection of neuroplastin in *Xenopus laevis* oocytes by RT-PCR

RNA was extracted from *Xenopus laevis* oocytes, liver and thymus using TRIzol® Reagent (Invitrogen) according to the manufacturer's protocol. cDNA was synthesised with Expand Reverse Transcriptase (Roche) and used in PCR reactions. The RT-PCR primers for basigin and embigin were the same as those used previously [18] while those for neuroplastin were forward (CGAGCCCAAGATCACTGCCACTGAGG) and backward (GCGTACACGCAAGATGGTGGTGCTGTTG). These corresponded to residues 139–164 and 691–718 of *Xenopus laevis* neuroplastin mRNA (GI: 28279875). Thermocycling was performed using the following parameters: 1 min at 95°C, 1 min at 55°C and 1 min at 72°C for 30 cycles. PCR products were analysed by agarose gel electrophoresis.

### MCT2 expression and measurement of transport activity in Xenopus laevis oocytes

cRNA for rat MCT2 was prepared and injected into *Xenopus laevis* oocytes as described previously [18]. For all assays 10 ng MCT cRNA±100 ng antisense RNA in 13.8 nl water were injected. MCT2 and embigin expression at the plasma membrane of oocytes was confirmed by immunofluorescence microscopy and Western blotting of membrane fractions as described previously [18], [20]. The antisense nucleotide against *Xenopus laevis* basigin (TTCTCATAAATAAAGATTATTGTG) was the same as used previously [18] whilst for neuroplastin the sequence used was (GATGGTGGTGCTGTTGTCTGAG) which corresponded to bases 228–254 of neuroplastin mRNA (GI: 28279875). Transport activity of MCT2 expressed in oocytes was determined by measuring the uptake of 0.5 mM [^14^C]-L-lactate for 5 min at room temperature as described previously [18].

### Animals

Animal procedures conformed to institutional regulations and the Guide to the Care and Use of Experimental Animals from the Canadian Council for Animal Care. Adult Sprague-Dawley rats (Charles River Laboratories, St. Constant, PQ) were used throughout. This study was specifically approved by the Royal Holloway institutional ethics committee. For immunohistochemistry, adult rats were deeply anesthetized with sodium pentobarbital (60 mg/kg i.p.) prior to transcardiac perfusion with normal saline, followed by either 4% paraformaldehyde/0.2% glutaraldehyde in 0.1M phosphate buffer (PB; pH 7.4) or Bouin's fixative [22]. The brains were removed and stored in fixative overnight at 4°C. The tissue was cryoprotected in 10% sucrose (2 hours), 20% sucrose (2 hours), and 30% sucrose (overnight) and cryosectioned at 20 µm in the transverse or sagittal planes. The sections were mounted on slides and allowed to dry overnight.

### Antibodies

Anti-zebrin II is a mouse monoclonal antibody produced by immunization with a crude cerebellar homogenate from the weakly electric fish *Apteronotus* [21]: it was used directly from spent hybridoma culture medium diluted 1∶400. Chicken anti-MCT2 is a polyclonal antipeptide antibody raised in chicken against the C-terminus of rat MCT2 and was obtained from Millipore (AB1287). It was used diluted 1∶100. Rabbit anti-neuroplastin is a polyclonal antibody raised in rabbit against all three neuroplastin Ig domains [4]. It was used diluted 1∶500.

For immunohistochemistry, all antibodies were diluted in blocking solution (0.1% Triton X-100, 10% normal goat serum in phosphate buffered saline (PBS), pH 7.4.

Replacement of the primary antisera with normal serum of the same species or by myeloma-conditioned culture medium resulted in no immunostaining.

### Immunocytochemistry

For double fluorescence immunohistochemistry, sections were incubated for 1 hour in blocking solution, then for 18 hours in primary antibody at room temperature, rinsed for 3×5 minutes in PBS, and incubated for 2 hours in CY2-conjugated rabbit anti-mouse secondary antibody (Jackson Immunoresearch Laboratories, West Grove, PA: diluted 1∶1,000 in blocking solution). Additional rinsing (3×5 minutes) was followed by incubation in the second primary antibody for 18 hours, rinsing in PBS and incubation for 2 hours in CY3-conjugated goat anti-chicken Ig antibody (Jackson Immunoresearch Laboratories Inc: diluted 1∶1,000 in blocking solution). Finally, the sections were rinsed in PBS and coverslipped using Fluorsave (Calbiochem, La Jolla, CA). Photomicrographs were captured with a Spot Cooled Color digital camera (Diagnostic Instruments Inc.). Montages were constructed in Adobe Photoshop (Adobe Systems, Mountain View, CA). The images were cropped and corrected for brightness and contrast but not otherwise manipulated.

Peroxidase immunohistochemistry was carried out on free-floating sections as described previously [22]. Briefly, tissue sections were washed thoroughly, blocked with 10% normal goat serum (Jackson Immunoresearch Laboratories, West Grove, PA) and then incubated in anti-MCT2 for 16–18 hours at room temperature. Finally, sections were washed and incubated in biotinylated goat anti-chicken Ig antibody (diluted 1∶1000 in blocking solution; Jackson Immunoresearch Laboratories, West Grove, PA) for 2 hours at room temperature, and binding revealed by using a Vectastain ABC staining kit with diaminobenzidine as the chromagen (Vector Laboratories Inc., Burlingame, CA). Sections were mounted on slides, dehydrated through an alcohol series, cleared in xylene and cover-slipped with Entellan mounting medium (BDH Chemicals, Toronto, ON).

### Statistical Analysis

The FRET data was analysed using an unpaired two tailed t test.

## Results

### Neuroplastin chaperones MCT2 to the plasma membrane

Although the neuroplastins play a key role in activity dependent long term synaptic plasticity and modulate neurite outgrowth other functions of these proteins have been unclear. A range of studies has shown that CD147/basigin and embigin bind to and are involved in trafficking members of the monocarboxylate transporter family to the cell surface [14], [15], [16], [18]. Basigin and embigin are required to chaperone MCTs1 and 4, and MCT2 respectively to the plasma membrane and must remain associated for MCTs to exhibit transport activity [16]. In the absence of a suitable ancillary protein, MCT1, MCT2 and MCT4 accumulate in the perinuclear region, but when co-transfected with either basigin (MCT1 and MCT4) or embigin (MCT2) they are correctly translocated to the plasma membrane [15], [16]. Therefore we have investigated whether neuroplastin can also associate with MCTs, and especially with MCT2 which is thought to be the major neuronal MCT in rodents [12], [23]. To test this hypothesis, COS-7 cells were transfected with MCT2 alone or together with either np55 or np65, on the basis that if the neuroplastins bind to MCT2, only co-transfection of cells with neuroplastin and MCT2 would result in a translocation of the transporter to the plasma membrane. COS-7 cells were used as they do not express endogenous np65, np55 or MCT2.

**Figure 1:**
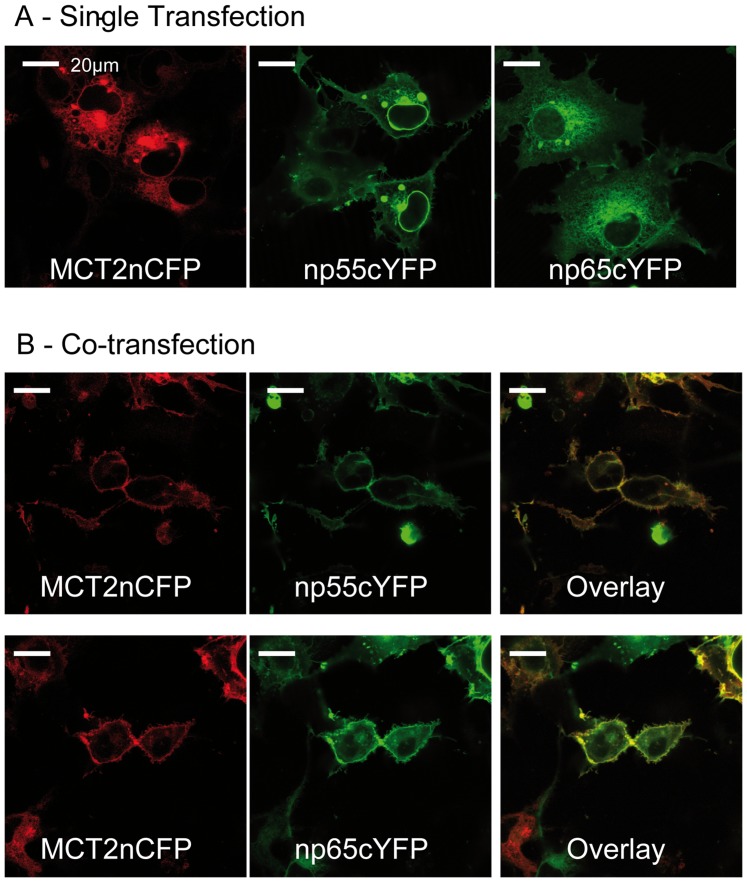
Co-transfection with np55 or np65 enables expression of MCT2 at the plasma membrane. Cos-7 cells were transiently transfected with MCT2 tagged at its N-terminus with CFP and/or np55 or np65 C-terminally labelled with YFP. The expression of np55 or np65 and MCT2 was detected by confocal microscopy. **A:** In single transfected cells MCT2 remains in the perinuclear region while neuroplastin is located at the plasma membrane and in the soma. **B and C:** In contrast, when MCT2 is transfected together with np55 or np65 both proteins are expressed at the cell surface. Cells were imaged using the Leica confocal imaging spectrophotometer system (TCS-SP2) attached to a Leica DMIRBE inverted epifluorescence microscope.

Single transfection of MCT2 tagged with cyan-fluorescent protein (CFP) showed no staining of the plasma membrane (Fig. 1A). The expressed proteins remained in the perinuclear compartment, which suggests their retention in the endoplasmic reticulum and Golgi apparatus. This is in agreement with previous results [15], [18]. Neuroplastins tagged with yellow-fluorescent protein (YFP) were expressed at the cell surface of singly transfected cells, but a considerable amount was still detected in the cytoplasm. However, when co-transfected with either np55 or np65 the majority of MCT2 was co-expressed with neuroplastin at the plasma membrane and only a small amount remained in the cytoplasm (Fig. 1 panel B). In the co-transfected cells most np55 and np65 was also detected at the plasma membrane rather than in the cytoplasm. Thus co-expression of MCT2 and np55 or np65 enables both proteins to be properly targeted to the cell surface consistent with their direct interaction, analogous to that shown for MCT1 with basigin and for MCT2 with embigin.

**Figure 2:**
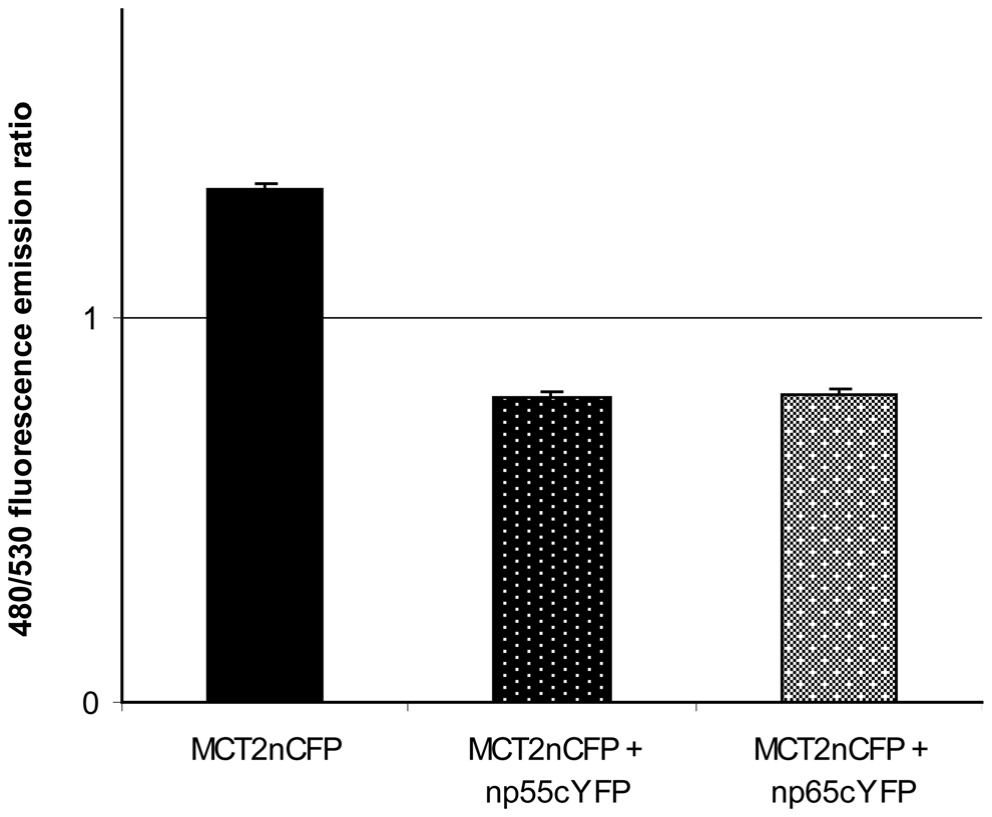
FRET measurements demonstrate a close association between MCT2 and np55 and np65. COS-7 cells were transiently co-transfected with the MCT2nCFP, np55cYFP and npc65-YFP constructs indicated (where n and c refer to the N- or C- terminal location of the CFP/YFP tag. Live cell imaging with determination of FRET was performed as described under “Methods”. Data are presented as means ± S.E.M. for 26, 22 and 26 separate cells respectively.

Fluorescence resonance energy transfer (FRET) was used to confirm a direct binding between the neuroplastins and MCT2. Np65 or np55 were C-terminally labelled with YFP and co-transfected with MCT2 tagged with CFP on its N-terminus. FRET was determined by measuring the 480∶530 nm fluorescence emission ratio when excited at 430 nm. When FRET occurs this ratio should be substantially less than that for MCT2-CFP expressed alone. It should be noted that optimal conditions for FRET have been established previously and a variety of techniques employed to confirm that the 530 nm emission does represent FRET mediated by MCT/ancillary protein interaction [16], [19]. The FRET data for the MCT2/np55 and np65 interactions are shown in Fig. 2. The mean 480∶530 nm fluorescence emission ratio value (± S.E.M) for MCT2-CFP alone was 1.33±0.016 (n = 26) while values for MCT2-CFP plus np55-YFP (n = 26) and MCT2-CFP plus np65-EYFP (n = 22) were 0.797±0.014 and 0.792±0.018 respectively. We also observed FRET when MCT2 was tagged with CFP on the C-terminus, which is also intracellular (data not shown). These data strongly suggest that np55 and np65 associate with MCT2 *in vivo* and are likely to play a role in translocation and localisation of MCT2 to the cell surface in some tissues.

### Knockdown of neuroplastin with antisense RNA inhibits MCT2 localisation to the cell surface and inhibits lactate uptake by *Xenopus* oocytes

**Figure 3:**
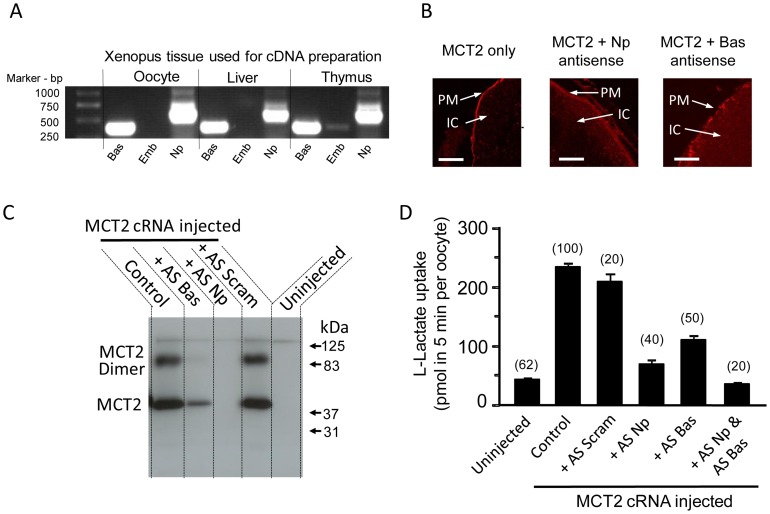
*Xenopus* oocytes express neuroplastin that facilitates MCT2 expression at the plasma membrane. **A:** shows the results of RT-PCR performed on mRNA extracted from *Xenopus laevis* oocytes, liver and thymus as described in the Experimental section. Primers for basigin (Bas), embigin (Emb) and neuroplastin (Np) were used as indicated. **B:** shows the expression of MCT2 in oocytes with or without antisense mRNA for basigin or neuroplastin detected by immunofluorescence microscopy. Antisense treatment reduces expression at the plasma membrane (PM) but increases intracellular expression (IC). The scale bar is 50 µm. **C:** shows parallel data for MCT2 expression in a crude membrane fraction derived from the oocyte and detected by SDS-PAGE and Western blotting. Here, scrambled (Scram) antisense RNA is used as an additional control. **D:** presents parallel data for the transport of [^14^C]-L-lactate into oocytes determined over 5 min. Error bars represent the S.E.M. of the number of oocytes indicated.

In a second set of experiments we investigated the role of neuroplastin in the expression of MCT2 in *Xenopus laevis* oocytes. Previously it has been shown that these oocytes do not express MCT2 but do express basigin and neuroplastin [18]. In Figure 3 (panel A) using RT PCR of oocyte cDNA with appropriate specific primers we confirm that the oocytes express basigin and also demonstrate the presence of neuroplastin. We then investigated the effect of neuroplastin knockdown using antisense RNA technology on surface expression of exogenous MCT2 in the oocytes injected with MCT2 cRNA. Immunofluorescence studies (Fig. 3, panel B) show that MCT2 is concentrated at the plasma membrane of control oocytes injected with MCT2 cRNA as expected. However, in oocytes treated with neuroplastin antisense RNA expression of MCT2 at the plasma membrane is barely detected above the diffuse background staining throughout the cell. By contrast, in oocytes treated with basigin antisense RNA the level of MCT2 at the plasma membrane was significantly reduced, but still detectable as immunoreactive punctae. Parallel data analysing the expression of MCT2 in a crude membrane fraction prepared from control (MCT cRNA injected only) and antisense-treated oocytes (Fig. 3, panel C) show no MCT2 in a membrane fraction from MCT2 expressing oocytes treated with antisense neuroplastin. Although the level of MCT2 in the membrane fraction is much reduced in oocytes injected with basigin antisense RNA, MCT2 is still detectable. This is consistent with the immunocytochemical data shown in panel B. The specificity of the antisense mRNA is shown by the fact that treatment with scrambled antisense RNA had no effect on the level of MCT2 present in the membrane fraction compared to control oocytes.

The functional consequences of the reduced transport and localisation of MCT2 at the oocyte plasma membrane was determined by measuring lactate uptake. The results (Fig. 3, panel D) show that in oocytes injected with MCT2 cRNA lactate transport was increased by some 500% compared to uninjected oocytes. Injection of oocytes with MCT2 and np55 cRNAs together did not further affect the rate of lactate uptake compared to oocytes injected with MCT2 cRNA alone. The uptake values expressed as pmol lactate uptake in 5 min were: non-injected oocytes, 32±3; MCT2 only injected oocytes, 296±24; MCT2 and Np55 injected oocytes, 294±15 (n = 10 for all groups). Treatment of oocytes expressing MCT2 with neuroplastin antisense RNA reduced lactate transport to a level only slightly greater than seen in native oocytes. Injection of basigin antisense RNA also lowered lactate transport compared to the control MCT2 RNA injected cells. However, consistent with the data shown in panels B and C, the effect was not as great as that seen for oocytes treated with neuroplastin antisense RNA. The level of lactate transport observed in cells co-treated with both neuroplastin and basigin antisense RNA was reduced to the background level observed in oocytes not injected with MCT2 cRNA. Again the specificity of the reduction is shown by the treatment with scrambled antisense RNA where there was no significant effect of the treatment on lactate transport compared to control (MCT2 cRNA injected) oocytes. These data clearly show that neuroplastins can play a key role in delivering MCT2 to the cell surface and in supporting lactate transport across the cell membrane. The data also confirm that basigin can act as an ancillary protein for MCT2 as well as MCT1, but that it is not as effective in this capacity as either neuroplastin or embigin.

### Neuroplastin and MCT2 co-localise in zebrin stripes in cerebellum

**Figure 4:**
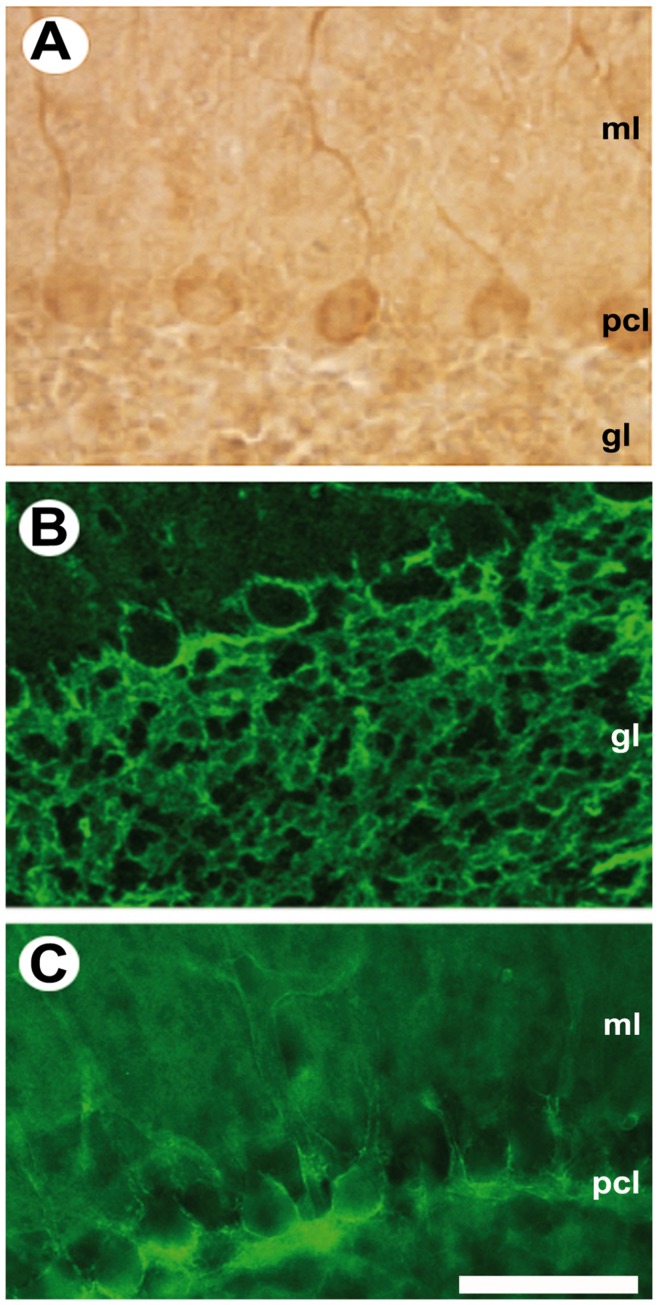
MCT2 immunoreactivity in 40 µm sagittal sections through the adult rat cerebellar cortex reveal Purkinje cell immunoreactivity. **A:** MCT2 immunoreactivity visualised by peroxidase reaction product is deposited in the Purkinje cell dendrites in the molecular layer (ml), Purkinje cell somata in the Purkinje cell layer (pcl), and weakly in the granular layer (gl). **B:** Anti-MCT2 immunofluorescence staining is prominent in the granular layer (gl). **C:** Anti-MCT2 immunofluorescence staining outlines Purkinje cell somata in the Purkinje cell layer (pcl) and their proximal dendrites in the molecular layer (ml): the distal dendritic arbour is either weakly stained or unreactive. Scale bar = 50 µm.

Given the potential importance of the neuroplastins in transporting MCT2 to the neuronal cell surface and to the synapse we have investigated their co-localisation in the cerebellum. Previous immunocytochemical studies have shown a neuronal localisation of MCT2 thoughout the brain [24]. In cerebellum MCT2 immunoreactivity is concentrated in Purkinje cells and in mossy fibres [24]. Since neuroplastin immunoreactivity is concentrated in parasagittal stripes in cerebellum we sought to establish if MCT2 immunoreactivity is colocalised in the same parasaggital stripes [22]. Figure 4 shows sagittal sections through the adult rat cerebellar cortex immunostained for MCT2. Peroxidase reaction product is deposited in the somata and dendrites of Purkinje cells (Fig. 4A). Consistent with previous descriptions [24]–[27] immunoreactivity is also prominent in the granular layer (Fig. 4B). The white matter tracts are unreactive (not shown). In the molecular layer, anti-MCT2 immunofluorescence reveals a delicate outlining of Purkinje cell somata and proximal dendrites (Fig. 4C). Staining was weak or absent in distal dendrites. Blood vessel staining was also prominent-also reported in [28].

**Figure 5:**
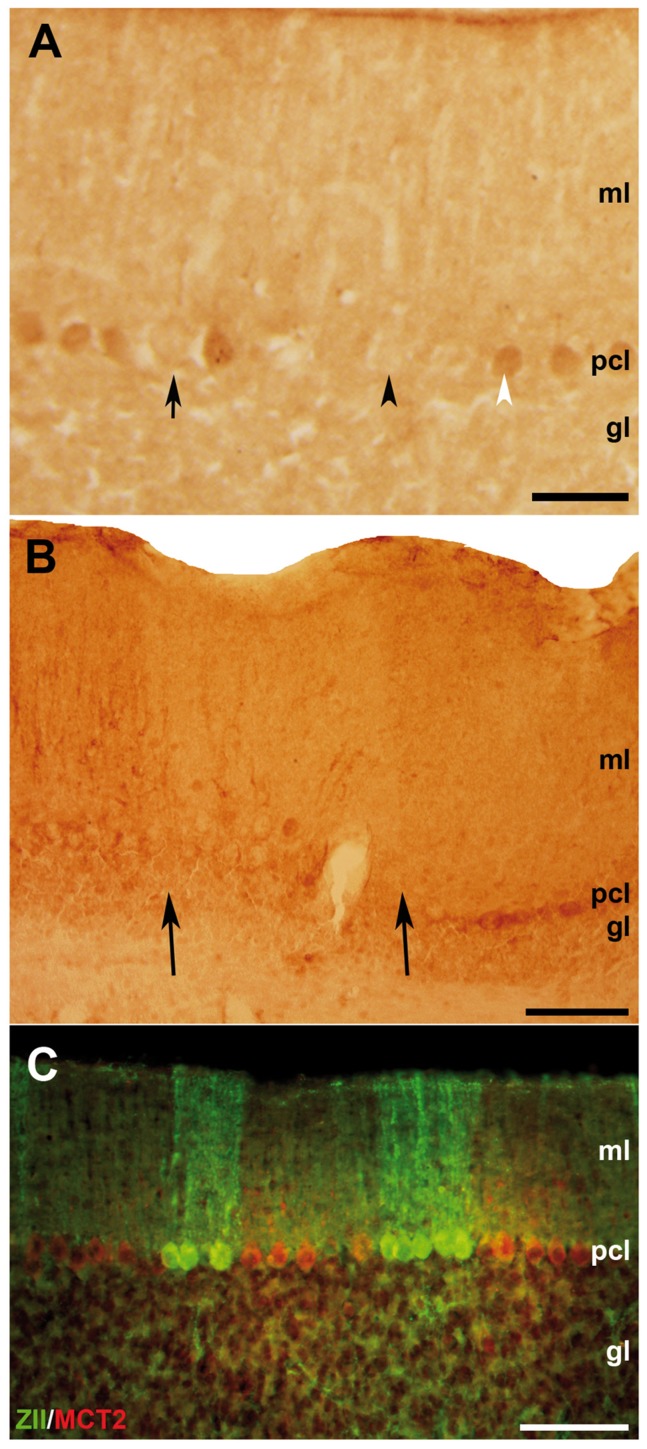
MCT2 is selectively expressed by Purkinje cell subsets. **A:** Levels of MCT2 immunoreactivity vary between Purkinje cell somata of the Purkinje cell layer (pcl) from strong (white arrowhead), to weak (black arrow), to none (black arrowhead). No boundaries are seen in the granular layer (gl). **B:** Clear boundaries are seen in the molecular layer (ml) between regions that express high levels of MCT2 immunoreactivity and others that are either weakly reactive or unreactive (arrows). **C:** A transverse section through lobule VIa of the adult rat cerebellar cortex double immunofluorescence labelled for zebrin II/aldolase C (green) and MCT2 (red), showing an array of alternating Purkinje cell stripes. The stripes of MCT2 immunoreactivity alternate with those of zebrin II/aldolase C. Scale bars: A and C = 50 µm, B = 100 µm.

It is well known that the cerebellum consists of multiple Purkinje cell subtypes arranged into parasagittal stripes. In this context it is interesting that not all Purkinje cells express MCT2 at the same level and runs of immunoreactive Purkinje cell somata are separated by others that are unstained (Fig. 5A). In some regions this alternation manifests as sharp boundaries in the molecular layer (Fig. 5B). The same is true for neuroplastin immunoreactivity, which is expressed in a reproducible array of parasagittal stripes in the adult cerebellar cortex [22] that alternate with stripes revealed by using anti-zebrin II/aldolase C [21], [29], [30], [31]. Unfortunately, neuroplastin immunostaining is only detected after Bouin's fixation [22], but Bouin's fixative abolishes MCT2 immunoreactivity. We have therefore been unable to compare the distributions of MCT2 and neuroplastin directly. As it has already been established that neuroplastin immunoreactivity localises to zebrin II negative stripes in adult cerebellum we have compared the heterogeneous distribution of MCT2 immunoreactivity directly to the distribution of zebrin II/aldolase C. Transverse sections were double immunofluorescence stained for MCT2 and zebrin II (Fig. 5C). We focused on lobule VIa of the vermis, where neuroplastin is expressed in a reproducible array of parasagittal zebrin II-immunonegative stripes [22]. The results show that MCT2 immunoreactivity in lobule VI is also preferentially restricted to the zebrin II-immunonegative (i.e., the neuroplastin-immunopositive) stripes. The co-enrichment of MCT2 and neuroplastin in the zebrin II negative stripes is consistent with a role for the neuroplastins as an accessory protein for MCT2 in cerebellar Purkinje cells.

## Discussion

### Neuroplastins directly bind to and support MCT2 function

It is well established that the two other members of the basigin group of the Ig superfamily besides the neuroplastins, basigin and embigin (gp70), play key roles as accessory proteins for the proton linked MCTs. Indeed basigin knockout mice are functionally blind due to the impairment of MCT function in the retina and retinal epithelium [32], [33]. MCT2 is thought to be a major neuronal monocarboxylate transporter in rodent brain and it has the highest affinity of the MCTs for lactate and pyruvate [34]. The present data unequivocally show that both np65 and np55 can act as accessory proteins for MCT2, chaperoning it to the cell surface and supporting its function in lactate transport. The data also show that it is preferred over basigin as the partner for MCT2. Previously embigin has been shown to be the preferred accessory protein for MCT2 in some tissues [16], [18]. Although embigin is strongly expressed in pre-implantation and early post implantation embryos its expression decreases during organogenesis and only low levels are observed in adult organs [35], [36]. Nevertheless, embigin is present in adult brain and is expressed in some neurones [37]. However, there are no published studies of the detailed regional, cellular and subcellular localisation of embigin in brain. The present results together with previous detailed studies of the localisation of the neuroplastins in rodent brain [4], [22] suggest that np55 and np65 are the main accessory proteins of MCT2, at least in some neuronal types and subpopulations. Furthermore, the developmental increase in neuroplastin expression coupled with the developmental decrease in embigin expression support a prominent role for neuroplastin as a preferred accessory protein for MCT2 in some neuronal populations in the adult rodent brain. Unfortunately, we have not been able to use co-immunprecipitation experiments from either brain homogenates or membrane preparations to provide further evidence for a neuroplastin-MCT2 interaction in brain for technical reasons. MCT2 antibodies do not work well in immunoprecipitation experiments and the level of neuroplastin is much higher than that for MCT2 in brain. Since neuroplastin interacts with a number of other binding partners including GABA_A_ receptor subunits and the FGF receptor [6], [8], [11] it is likely that only a small fraction of the total neuroplastin is associated with MCT2. Thus the level of MCT2 co-immunpreciptated by neuroplastin antibodies is below the limit of detection.

### Significance of Neuroplastin and MCT2 co-localisation in Purkinje cells and in zebrin stripes in cerebellum

Both MCT2 and neuroplastin are expressed in Purkinje cells. The distribution described here is supported by previous immunocytochemical studies [16], [24], [27], [38], [39] and is also consistent with the MCT2 mRNA distribution revealed by using *in situ* hybridization, e.g. [40]. It is clear that MCT2 is associated with the Purkinje cell surface *in vivo*. Although it is not obviously associated with spines (parallel fibre-Purkinje cell synapses) in our material, this association has been shown by electron microscopy immunogold techniques [38].

Strikingly, not all Purkinje cells show MCT2 immunoreactivity. In some cases, we have shown that MCT2 expression is restricted to the same Purkinje cell (zebrin II negative) stripes that also preferentially express neuroplastin. However, this is not an obligatory association and we also see MCT2 immunoreactivity in cerebellar regions in which neuroplastin is more weakly expressed. These data again suggest both embigin and the neuroplastins may be MCT2 accessory proteins in different neuronal types and subpopulations.

The evidence that MCT2 is found preferentially in neuroplastin-immunoreactive Purkinje cell subsets, and that it can be co-localized with glutamate receptors in the postsynaptic densities of parallel fiber-Purkinje cell synapses [38], is additional evidence that synaptic transmission differs between Purkinje cell stripes. Differences between zebrin II+/neuroplastin− and zebrin II−/neuroplastin+ stripes include both functional differences, e.g. [41], and the differential expression of a suite of molecules associated with glutamatergic neurotransmission and long-term depression (LTD) of synaptic efficacy at both parallel fibre and climbing fibre synapses, e.g. [42]. These include the metabotropic glutamate receptor [43], excitatory amino acid transporter 4 [44], an inositol 1,4,5-trisphosphate (IP3) receptor [45], phospholipase Cβ3/4 [46], and protein kinase C [47]. The preferential expression of MCT2 by neuroplastin-immunoreactive Purkinje cell subsets is a further reflection of this heterogeneity. These data suggest that in addition to the established role of neuroplastins in regulating the surface expression of GluR1 receptors in hippocampal LTP [4], [10] they may also play a similar role in LTD between parallel fibre and Pukinje cells synapses in cerebellum. Many molecules implicated in cerebellar LTD are expressed in parasagittal stripes in subsets of Purkinje cells-for reviews see [48], [49]. Importantly these include molecules involved in energy generation (e.g., zebrin II/aldolase C – [30]) and now MCT2. This raises the possibility that subsets of Purkinje cells preferentially use lactate as an energy source.

Lactate is the main monocarboxylate found in the adult brain. Various reports show that lactate, mainly released by astrocytes, is used as an important energy substrate by neurons and sustains neuronal activity, including action potential propagation, reviewed in [13]. MCT2 was detected at the surface and in the cytoplasm of the cell bodies as well as on the membrane of dendrites [13]. MCT2 appears to be present also on many axonal projections. Most importantly, MCT2 is present postsynaptically, with high levels expressed in the PSD where it co-localises with δ2-glutamate receptors as well as the AMPA receptor GluR2/3 [38], [50], [51]. It was also detected within the dendritic spine, forming an intracellular pool. Therefore it was suggested that, like the AMPA receptor, MCT2 could undergo a process of translocation and endocytosis at the PSD. Additionally the expression of MCT2 concomitant with np65 correlates with synaptogenesis in neurons in culture [52], which suggest a possible link with the development of synapses and/or synaptic activity. After stimulation with the neurotransmitter noradrenaline MCT2 expression is enhanced [53]. Taken together, the postsynaptic localisation of MCT2 at the synapse could ensure adequate supply of lactate as a respiratory fuel to energise the neuron after stimulation. Indeed Suzuki et al. [54] demonstrated the importance of astrocyte-neuron lactate transport in long term memory formation and in LTP. They also show that disrupting the expression of MCT1 and MCT4, both of which are expressed in astrocytes, leads to amnesia, but this can be rescued by lactate, but not glucose. While disruption of the neuronally expressed MCT2 also causes amnesia, significantly this cannot be rescued by lactate. In this regard our previous studies have shown that np65 is recruited into the PSD following paradigms which result in long term activity dependent synaptic plasticity including LTP and kainate seizures [4]. Reports that lactate is an important energy substrate at the synapse at times of high energy demand coupled with the observations that np65 can be rapidly translocated in to the PSD in response to sustained increases in synaptic activity are consistent with np65 (and np55 in cerebellum) transporting MCT2 to the post synaptic region and supporting lactate transport to the nerve terminal under conditions of high energy demand.

In conclusion the following observations strongly support the hypothesis that neuroplastins play a physiological role as the accessory protein for MCT2 at least in some neuronal populations: 1) neuroplastins bind to and support MCT2 function in transfected cell lines; 2) neuroplastins directly bind to MCT2; 3) the low level of embigin and high level of neuroplastin in adult brain, and 4) the co-localisation of neuroplastins and MCT2 in zebrin stripes in cerebellum.
